# Value of American Thoracic Society Guidelines in Predicting Infection or Colonization with Multidrug-Resistant Organisms in Critically Ill Patients

**DOI:** 10.1371/journal.pone.0089687

**Published:** 2014-03-19

**Authors:** Jianfeng Xie, Xudong Ma, Yingzi Huang, Min Mo, Fengmei Guo, Yi Yang, Haibo Qiu

**Affiliations:** 1 Department of Critical Care Medicine, Zhong-Da Hospital, School of Medicine, Southeast University, Nanjing, China; 2 School of Medicine and Health Management, Tongji Medical College of Huazhong University of Science and Technology, Wuhan, China; Charité, Campus Benjamin Franklin, Germany

## Abstract

**Background:**

The incidence rate of infection by multidrug-resistant organisms (MDROs) can affect the accuracy of etiological diagnosis when using American Thoracic Society (ATS) guidelines. We determined the accuracy of the ATS guidelines in predicting infection or colonization by MDROs over 18 months at a single ICU in eastern China.

**Methods:**

This prospective observational study examined consecutive patients who were admitted to an intensive care unit (ICU) in Nanjing, China. MDROs were defined as bacteria that were resistant to at least three antimicrobial classes, such as methicillin-resistant *Staphylococcus aureus* (MRSA), vancomycin-resistant *enterococci* (VRE), *Pseudomonas aeruginosa, Acinetobacter baumannii*. Screening for MDROs was performed at ICU admission and discharge. Risk factors for infection or colonization with MDROs were recorded, and the accuracy of the ATS guidelines in predicting infection or colonization with MDROs was documented.

**Results:**

There were 610 patients, 225 (37%) of whom were colonized or infected with MDROs at ICU admission, and this increased to 311 (51%) at discharge. At admission, the sensitivity (70.0%), specificity (31.6%), positive predictive value (38.2%), and negative predictive value (63.5%), all based on ATS guidelines for infection or colonization with MDROs were low. The negative predictive value was greater in patients from departments with MDRO infection rates of 31–40% than in patients from departments with MDRO infection rates of 30% or less and from departments with MDRO infection rates more than 40%.

**Conclusion:**

ATS criteria were not reliable in predicting infection or colonization with MDROs in our ICU. The negative predictive value was greater in patients from departments with intermediate rates of MDRO infection than in patients from departments with low or high rates of MDRO infection.

**Trial Registration:**

ClinicalTrials.gov NCT01667991

## Introduction

Sepsis is a common cause of morbidity and mortality in critically ill patients [1], [2]. The use of broad-spectrum antibiotics has led to a worldwide increase of multidrug-resistant organisms (MDROs) in intensive care units (ICUs) [3]–[5], and this is an increasing challenge for ICU physicians [6]–[8]. Previous studies indicated that inappropriate initial antibiotic treatment can increase the risk of mortality, and that effective antimicrobial administration within the first hour of documented hypotension is associated with improved survival to hospital discharge in adult patients with septic shock [9], [10]. Therefore, it is critically important to initiate an appropriate initial antibiotic treatment based on early diagnosis [11], [12].

In 2005, the American Thoracic Society (ATS) published guidelines [13] for treatment of patients with healthcare-associated pneumonia. These guidelines recommend selection of an initial empiric therapy based on the presence of risk factors for MDROs. Although the guidelines were developed for management of patients with pneumonia, the risk factors for infection with an MDRO are similar for patients with other types of infections, such as bloodstream infection [14]. Thus, the MDRO-related risk factors identified in the ATS guidelines may be helpful in guiding initial antibiotic treatment of such patients. Some research has demonstrated that mortality due to severe sepsis can be reduced when such patients are managed according to the ATS guidelines [15], [16].

However, the accuracy of the ATS guidelines in predicting infection or colonization by MDROs is unclear. A recent study [17] indicated that the ATS guidelines had low specificity and positive predictive value. Therefore, if patients with severe sepsis and septic shock receive antibiotic treatment according to the ATS guidelines, many of them will be over-treated with broad-spectrum antibiotics.

Different regions may have different proportions of patients with infection or colonization by MDROs, so the value of the ATS guidelines may be different in different countries [18]. Even within the same hospital, the proportion of MDROs may differ among different departments. To our knowledge, there have been no reports on use of the ATS guidelines in predicting infection or colonization with MDROs in critically ill patients in China. This prospective observational trial was designed to identify the value of the ATS guidelines in predicting infection or colonization with MDROs in critically ill patients based on culture results of nasal swabs, secretions of the lower respiratory tract, location of the infection, and risk factors for infection by an MDRO. We hypothesized that usefulness of the ATS guidelines in our center differs from that in Western counties and also differs for patients from different departments within our hospital.

## Patients and Methods

### Study Design

This prospective observational study was performed in a 20-bed ICU in a university-affiliated hospital from April 2010 to September 2011. The protocol was approved by the Institutional Ethics Committee of Zhong-Da Hospital (Approval Number: 2011ZDLL012.0). Each patient (or designated proxy) provided written informed consent for use of clinical data, and the ethics committee approved our consent procedure. Participation in the study did not necessitate any changes in treatment. All patients hospitalized in the ICU were eligible. Some ICU patients were transferred from other departments within our hospital. Bacterial cultures of nasal swabs, secretions of the lower respiratory tract, and other locations were performed at ICU admission and discharge. Specimens from other sources such as blood, urine, other body fluids were also cultured according the patient’s clinical status. Screening for MDROs was performed in our ICU as part of an infection control policy, and microbiological cultures of other specimens were performed according to the patient’s clinical status. There was a mean of 1.48 samples per patient. The antimicrobial treatment protocol for treatment of severe sepsis and septic shock were according to the recent ATS recommendations.

### Data Collection and Definitions

All data concerning patient characteristics at ICU admission and during the ICU stay were prospectively collected. MDROs were defined as bacteria that were resistant to at least three antimicrobial classes, including methicillin-resistant *Staphylococcus aureus* (MRSA), vancomycin-resistant *enterococci* (VRE), *Pseudomonas aeruginosa*, *Acinetobacter baumannii*, and extended-spectrum β-lactamase (ESBL)-producing Gram-negative bacilli based on laboratory testing. Determination of the minimal inhibitory concentration (MIC) was used to evaluate drug resistance in accordance with the standards set for European microorganisms (European Committee on Antimicrobial Susceptibility Testing. Breakpoint Tables for Interpretation of MICs and Zone Diameters, Version 1.3, 2011. http://www.eucast.org/clinical_breakpoints). MRSA was defined as *Staphylococcus aureus* resistant to oxacillin, and VRE was defined as *enterococci* resistant to vancomycin. ESBL-producing organisms were defined as Gram-negative bacilli resistant to ceftazidime but sensitive to an enzyme inhibitor such as piperacillin–tazobactam. Resistance was only determined one time, even if there were duplicate isolates from same patient.

Colonization was defined as positive results in culture, but no indication of infection based on symptoms, signs, and imaging results. Infection was defined by the attending physician according the patient’s condition. Pneumonia was defined by the presence of new or progressive radiographically documented infiltrates with two of the following: *(i)* body temperature greater than 38.5°C or less than 36.5°C; *(ii)* leukocyte count greater than 10 000/μL or less than 4000/μL; and *(iii)* purulent sputum or tracheal aspirate. Other infections were defined according to the modified CDC criteria [19].

The identification of risk factors for infection by an MDRO were according to ATS guidelines [13]: current hospitalization of 5 days or more, prior antibiotic therapy, prior hospitalization, residence in a nursing home or extended-care facility, home infusion therapy within 30 days, chronic dialysis within 30 days, home wound care, family member with an MDRO, and immunosuppression. However, among the 610 included patients, 2 patients were residents in nursing homes or extended-care facilities, 10 patients received dialysis within 30 days, and 11 patients had home wound care. Prior antibiotic treatment was defined as use of any antibiotic during the 3 months preceding ICU admission. Prior hospitalization was defined as hospitalization for 2 or more days during the preceding 3 months.

All infected patients were treated by the attending physicians, and the choice and adjustment of antibiotics were in accordance with ATS guidelines. If a patient had a suspected community-acquired infection, an antibiotic such as penicillin, cefuroxime, or levofloxacin was selected. If a patient had a suspected hospital-acquired or healthcare-associated infection and no risk that it was an MDRO, an antibiotic such as ceftriaxone, levofloxacin, or moxifloxacin was selected. If a patient had a suspected hospital-acquired or healthcare-associated infection with a risk that it was an MDRO, an antibiotic such as ceftazidime, β-lactam/β-lactamase inhibitor, carbepenem plus antipseudomonal fluoroquinolone, or aminoglycoside was selected. If a patient had a suspected a MRSA infection, linezolid, vancomycin, or teicoplanin was selected.

### Statistical Methods

All statistical analyses were conducted with SPSS 13.0 software (release 13.0; SPSS, Chicago, IL). Continuous variables are presented as means and standard deviations (SDs) if they had normal distributions, and as medians and interquartile ranges (IQRs) if they had a non-normal distribution. Categorical variables are presented as counts and percentages. Differences between group means were analyzed by an independent two-sample *t*-test for continuous variables, the Mann-Whitney test for group median, and the Chi-square test or Fisher’s exact test with Yate’s correction if any cell number was less than five or close to zero (categorical variables). The sensitivity, specificity, positive predictive value (PPV), and negative predictive value (NPV) (defined below), were used to predict colonization or infection by MDROs at ICU admission. The accuracy of the ATS guidelines in predicting infection or colonization with MDROs at ICU admission was evaluated in all patients, and in certain subgroups of patients. If a patient had one or more risks, as defined above, the patient was considered to have increased risk for colonization or infection by an MRDO. Finally, a logistic regression model was used to identify risk factors for MDROs at ICU admission.

Sensitivity was defined as the proportion of positive risk factors in patients correctly identified as colonized or infected by an MRDO. Specificity was defined as the proportion of absent risk factors in patients that were not colonized or infected by an MDRO. PPV was defined as the proportion of MDRO colonized or infected patients considered at risk relative to all patients with risk factors. The NPV is the proportion of MDRO-free patients not considered at risk relative to all patients without risk factors.

## Results

**Figure 1:**
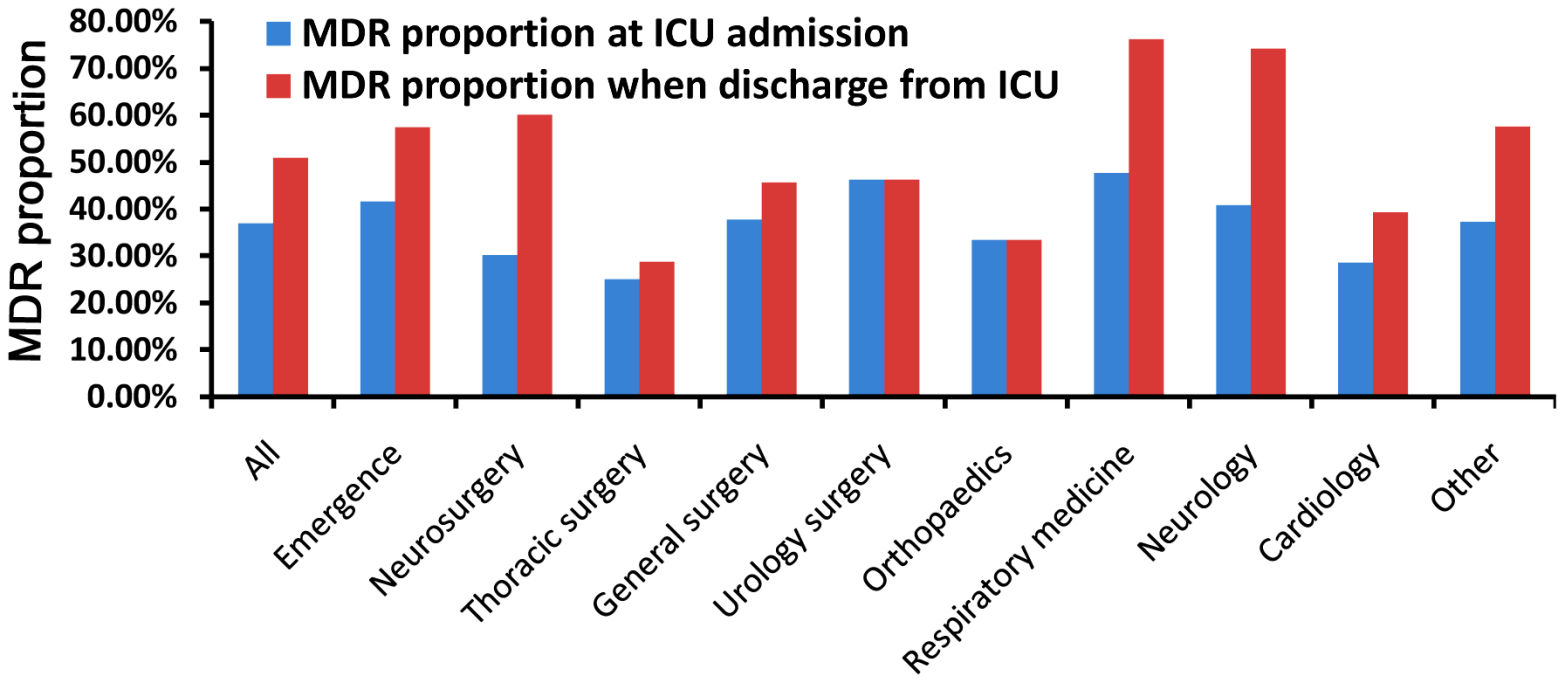
Proportion of patients with infection or colonization by an MDRO in different departments at admission (blue) and discharge (red).

A total of 610 patients were enrolled in this study, 225 (37%) of whom were colonized or infected with an MDRO at ICU admission, and 311 (51%) of whom were colonized or infected with an MDRO at ICU discharge. Figure 1 and Table S1 show the proportion of patients from the different departments. Table 1 shows the baseline characteristics of the 610 patients. The median duration of ICU stay was 5 days (IQR: 2–10 days), and the median length of hospital stay was 20 days (IQR: 10–37 days). One hundred sixty-one patients (26%) died in the ICU, corresponding to a mortality rate of 31%. Comparison of patients infected with MRDOs and non-MRDOs indicated that these two groups had similar age, gender, APACHE II score, admission source, number of dysfunctional organs, mechanical ventilation utilization, ICU mortality, and hospital mortality (Table 1). Among all 610 patients, 344 patients (56%) were diagnosed with an infection; among these 344 patients, 286 (83%) were diagnosed with pneumonia. Other infection sites included blood (n = 20, 5.8%), urinary system (n = 10, 2.9%), abdomen (n = 28, 8.1%), and others (n = 27, 7.8%). The presence of other diseases had no influence on therapy. All antibiotics were selected according to ATS guidelines, and all other treatments were performed according to Surviving Sepsis Campaign guidelines.

**Table 1:** Demographic and clinical characteristics of enrolled patients.

	Overall	MDRO	Non-MDRO	*P*-value
	(n = 610)	(n = 230)	(n = 380)	
**Age (years)^1^**	63.3±19.2	62.7±19.2	63.6±19.2	0.569 ^§^
**Gender^2^**				0.731 ^†^
Female	232 (38.0%)	85 (37.0%)	147 (38.7%)	
Male	378 (62.0%)	145 (63.0%)	233 (61.3%)	
**APACHE II score^1^**	15.9±8.4	16.0±8.1	15.9±8.6	0.925 ^§^
**Admission source^2^**				0.539^†^
Emergence	175 (28.7%)	72 (31.3%)	103 (27.1%)	
Surgery	295 (48.4%)	107 (46.5%)	188 (49.5%)	
Medicine	140 (23.0%)	51 (22.2%)	89 (23.4%)	
**Number of dysfunctional organs^1^**	2.0±1.3	2.0±1.3	2.0±1.4	0.837 ^§^
**Death in ICU^2^**				0.806 ^†^
Yes	161 (26.4%)	62 (27.0%)	99 (26.1%)	
No	449 (73.6%)	168 (73.0%)	281 (73.9%)	
**Death in Hospital^2^**				0.820 ^†^
Yes	189 (31.0%)	70 (30.4%)	119 (31.3%)	
No	421 (69.0%)	160 (69.6%)	261 (68.7%)	
**Duration of ICU stay^3^**	5.0 (2.0, 11.0)	5.0 (2.0, 10.0)	5.0 (2.0, 11.0)	0.690 ^‡^
**Duration of hospitalization^3^**	20.0 (10.0, 37.0)	19.0 (10.0, 36.0)	20.5 (9.8, 39.0)	0.581 ^‡^
**Mechanical ventilation^2^**				0.195 ^†^
Yes	470 (77.2%)	171 (74.3%)	299 (78.9%)	
No	139 (22.8%)	59 (25.7%)	80 (21.1%)	
**Ventilation days^3^**	2.0 (1.0, 5.5)	2.0 (1.0, 5.0)	1.0 (0, 6.0)	0.615 ^‡^
**Infection^2^**				0.774 ^†^
Yes	344 (56.4%)	128 (55.7%)	216 (56.8%)	
No	266 (43.6%)	102 (44.3%)	164 (43.2%)	

Data are presented as ^1^mean and standard deviation, ^2^count and percentage, or ^3^median and interquartile range.

*P*-values were calculated by ^§^two-sample *t*-test; ^†^chi-square test; and ^‡^Mann-Whitney test.

Abbreviations: MDR, multidrug-resistant; ICU, intensive-care unit.

**Figure 2:**
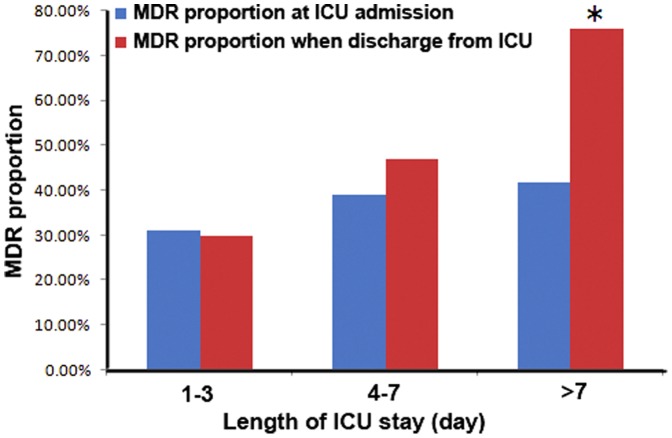
Proportion of patients who had ICU stays of 1–3 days, 4–7 days, and more than 7 days with infection or colonization by an MDRO at ICU admission (blue) and discharge (red). *Significant difference (*p*<0.001).

The number of patients colonized or infected with an MDRO increased from 225 (37%) at ICU admission to 310 (51%) at ICU discharge (*p*<0.001). There were similar increases for patients from the Departments of Emergency (41% to 57%, *p* = 0.003), Neurosurgery (30% to 60%, *p* = 0.020), Neurology (41% to 74%, *p* = 0.013), and others (37% to 57%, *p* = 0.006) (Figure 1). There were no significant changes in the proportion of patients with MDROs at admission and discharge whose ICU stays were 1–3 days (admission: 31%, discharge: 30%, p = 0.764) and 4–7 days (admission: 39%, discharge: 47%, p = 0.138). However, the change was significant for patients who stayed in the ICU for more than 7 days (admission: 42%, discharge: 76%, *p*<0.001) (Figure 2).

Table 2 shows the microorganisms associated with colonization or infection at admission and discharge. Among the 230 MDROs present at admission, 68 (28.6%) were MRSA and 66 (27.7%) were A. *baumannii*. At discharge, the rate of MRSA infection decreased to 16.1%, but the rate of A. *baumannii* infection increased to 48.2%.

**Table 2:** Infectious microorganisms identified at ICU admission and discharge.

Type of microorganism	ICU admission	ICU discharge
	n (%)	n (%)
**All**	823 (100)	800 (100)
**MDRO** 1	238 (28.9)	367 (45.9)
Methicillin-resistant *Staphylococcus aureus* 2	68 (28.6)	59 (16.1)
Vancomycin-resistant enterococci^2^	5 (2.1)	4 (1.1)
* Pseudomonas aeruginosa* 2	16 (6.7)	20 (5.4)
* Acinetobacterbaumannii* 2	66 (27.7)	177 (48.2)
* Escherichia coli* 2	18 (7.6)	20 (5.4)
* Klebsiellaoxytoca* 2	14 (5.9)	25 (6.8)
* Enterobacter cloacae* 2	7 (2.9)	3 (0.8)
* Proteus* species^2^	1 (0.4)	4 (1.1)
Other^2^	43 (18.1)	55 (15.0)
**Non-MDRO** 1	585 (72)	433 (54.1)
* Staphylococcus epidermidis* 3	288 (49.2)	208 (48.0)
* Staphylococcus haemolyticus* 3	25(4.3)	26(6.0)
Methicillin-sensitive S*taphylococcus aureus* 3	34(5.8)	27(6.2)
* Enterococcus* species^3^	18(3.1)	12 (2.8)
* Pseudomonas aeruginosa* 3	5 (0.9)	6 (1.4)
* Acinetobacterbaumannii* 3	10 (1.7)	10(2.3)
* Escherichia coli* 3	18 (3.1)	12(2.8)
* Klebsiellaoxytoca* 3	49(8.4)	28(6.5)
* Enterobacter cloacae* 3	10 (1.7)	9 (2.1)
* Proteus* species^3^	5(0.9)	4 (0.9)
Others^3^	123(21.0)	91(21.0)

Data are presented as counts and percentages.

1 indicated percentage is the count divided all microorganisms.

2 indicated percentage is the count divided multidrug-resistant bacteria.

3 indicated percentage is the count divided non-multidrug-resistant bacteria.

Table 3 shows the antibiotic treatments of patients during their ICU stays. One hundred and six patients (17.4%) did not receive antibiotics and 91 patients (14.9%) received combined antibiotic treatment at ICU admission. Among patients treated with antimicrobial agents, 463 patients (91.9%) were treated with antibacterial agents and 41 patients (8.1%) were treated with antifungal agents.

**Table 3:** Antibiotics used by patients during their ICU stays.

Antibiotic classification	N (%)
**Number of antibiotics**	
None	106 (17.4)
One	413 (67.7)
More than one	91 (14.9)
**Antibacterialagents**	
Penicillin	9 (1.5)
Cefuroxime	53 (8.7)
Ceftriaxone	42 (6.9)
Ceftazidime	29 (4.8)
Cefperazone-Sulbactam (Sulperzone)	168 (27.5)
Piperacilintazobactam	103 (16.9)
Imipenemcystatin	62 (10.2)
Meropenem	11 (1.8)
Levofloxacin	16 (2.6)
Moxifloxacin	11 (1.8)
Vancomycin	12 (2.0)
Teicoplanin	5 (0.8)
Linezolid	11 (1.8)
Other	15 (2.5)
**Antifungalagents**	
Fluconazole	28 (4.6)
Itraconazole	3 (0.5)
Voriconazole	2 (0.3)
Echinocandins	7 (1.1)
Amphptericin	1 (0.2)

Table 4 shows the risk factors for colonization or infection with bacteria at ICU admission according to ATS guidelines. A total of 421 patients (69.0%) had at least one risk factor at ICU admission. There were no significant differences for patients with MDRO and non-MDRO infections in terms of MDRO risk factors (70.0% *vs.* 69.0%, *p* = 0.683), prior antimicrobial treatment (53.5% *vs. *49.5%, *p* = 0.338), duration of hospitalization over 5 days before ICU admission (62.2% *vs.* 54.2%, *p* = 0.054), prior hospitalization (22.2% *vs.* 18.2%, *p* = 0.227), and immunosuppression (12.6% *vs.* 13.4%, *p* = 0.773).

**Table 4:** Risk factors for infection by MDROs according to ATS guidelines.

	Overall	MDRO	Non-MDR	*P*-value
	(n = 610)	(n = 230)	(n = 380)	
**Patients with risk factor**				0.683
Yes	421 (69.0%)	161 (70.0%)	260 (68.4%)	
No	189 (31.0%)	69 (30.0%)	120 (31.6%)	
**Prior antimicrobial treatment**				0.338
Yes	311 (51.0%)	123 (53.5%)	188 (49.5%)	
No	299 (49.0%)	107 (46.5%)	192 (50.5%)	
**Duration of hospitalization over 5 days before ICU admission**				0.054
Yes	349 (57.2%)	143 (62.2%)	206 (54.2%)	
No	261 (42.8%)	87 (37.8%)	174 (45.8%)	
**Prior hospitalization**				0.227
Yes	120 (19.7%)	51 (22.2%)	69 (18.2%)	
No	490 (80.3%)	179 (77.8%)	311 (81.8%)	
**Immunosuppression**				0.773
Yes	80 (13.1%)	29 (12.6%)	51 (13.4%)	
No	530 (86.9%)	201 (87.4%)	329 (86.6%)	

Data are presented as counts and percentages.

*P*-values were obtained by a chi-square test.

Table 5 shows the accuracy of the ATS guidelines in predicting colonization or infection with MDROs at ICU admission overall (n = 610), in patients with any infection (n = 344), and in patients with pneumonia (n = 286). In all cases, the sensitivity, specificity, NPV, and PPV were low. Among the 610 patients, 421 (69%) had at least one risk factor for an MDRO infection according to the ATS guidelines, and 301 (71%) of these patients used an antibiotic in the preceding 90 days. Among the 344 patients with an infection, 250 (73%) had at least one risk factor for an MDRO according to the ATS guidelines, as did 212 (74%) of the 286 pneumonia patients.

**Table 5:** Accuracy of ATS criteria in predicting colonization or infection by MDROs at ICU admission.

	Overall	Infection	Pneumonia
	(n = 610)	(n = 344)	(n = 286)
Sensitivity	161/230 (70.0%)	99/128 (77.3%)	81/105 (77.1%)
Specificity	120/380 (31.6%)	65/216 (30.1%)	50/181 (27.6%)
PPV	161/421 (38.2%)	99/250 (39.6%)	81/212 (38.2%)
NPV	120/189 (63.5%)	65/94 (69.1%)	50/74 (67.6%)

Abbreviations: PPV, positive predictive value; NPV, negative predictive value.

Table 6 shows the accuracy of the ATS guidelines in predicting colonization or infection with MDROs at ICU admission in patients came from different departments. The NPV was higher for patients from a medicine or surgery department (71.9% and 65.3%) than for patients from the Emergency Department (55.4%).

**Table 6:** Accuracy of ATS criteria in predicting colonization or infection by MDROs at ICU admission in patients from different departments.

	Emergency	Surgery	Medicine	*P*-value
	(n = 175)	(n = 295)	(n = 140)	
**Patient characteristic**				
Age (years)	61.3±21.6	61.8±18.2	69.0±17.0	<0.001† ‡
Gender				0.677
Female	62 (35.4%)	114 (38.6%)	56 (40.0%)	
Male	113 (64.6%)	181 (61.4%)	84 (60.0%)	
APACHE II score	17.1±7.5	13.4±8.2	19.6±8.5	<0.001§ † ‡
Patients with risk factor				0.053
Yes	119 (68.0%)	194 (65.8%)	108 (77.1%)	
No	56 (32.0%)	101 (34.2%)	32 (22.9%)	
Number of dysfunctional organs	2.6±1.3	1.4±1.2	2.5±1.2	<0.001§ ‡
Death in ICU				
Yes	61 (34.9%)	44 (14.9%)	56 (40.0%)	<0.001
No	114 (65.1%)	251 (85.1%)	84 (60.0%)	
Death in Hospital				<0.001
Yes	72 (41.1%)	54 (18.3%)	63 (45.0%)	
No	103 (58.9%)	241 (81.7%)	77 (55.0%)	
**Predictive value**				
Sensitivity	47/72 (65.3%)	72/107 (67.3%)	42/51 (82.4%)	
Specificity	31/103 (30.1%)	66/188 (35.1%)	23/89 (25.8%)	
PPV	47/119 (39.5%)	72/194 (37.1%)	42/108 (38.9%)	
NPV	31/56 (55.4%)	66/101 (65.3%)	23/32 (71.9%)	

§ indicates significant difference between patients from emergency and surgery departments.

† indicates significant difference between patients from emergency and medicine departments.

‡ indicates significant difference between patients from surgery and medicine departments.

Table 7 shows a comparison of the accuracy of the ATC guidelines in patients from departments with 3 different proportions of MDROs (≤30%, 31–40%, and >40%). The NPV was higher in patients when the proportion of MDROs was 31–40% (NPV = 69.1%) than when the proportion of MDROs was 30% or less (NPV = 60.4%) or more than 40% (NPV = 60.3%).

**Table 7:** Accuracy of ATS criteria in predicting colonization or infection by MDROs at ICU admission in patients from departments with different proportions of MRDOs.

MDR proportion	≤30%	31∼40%	>40%
	(n = 138)	(n = 223)	(n = 249)
Sensitivity	35/54 (64.8%)	57/78 (73.1%)	69/98 (70.4%)
Specificity	29/84 (34.5%)	47/145 (32.4%)	44/151 (29.1%)
PPV	35/90 (38.9%)	57/155 (36.8%)	69/176 (39.2%)
NPV	29/48 (60.4%)	47/68 (69.1%)	44/73 (60.3%)

Abbreviation: MDR, multidrug-resistant; ATS, American Thoracic Society; ICU, intensive-care unit; PPV, positive predictive value; NPV, negative predictive value.

Finally, Table 8 shows the results of a logistic regression analysis of risk factors associated with MDRO infection at ICU admission. The results indicate no significant association of infection with an MDRO at ICU admission and any of the tested variables.

**Table 8:** Logistic regression analysis for infection by MDROs at ICU admission.

	OR	95% CI	*P*-value
**Age (years)**	0.997	0.988, 1.007	0.562
**Gender**			
Female	Reference		
Male	1.086	0.766, 1.541	0.642
**APACHE II score**	1.003	0.980, 1.027	0.786
**Admission source**			
Emergency	Reference		
Surgery	0.881	0.565, 1.373	0.575
Medicine	0.832	0.518, 1.335	0.445
**Number of dysfunctional organs**	0.984	0.828, 1.171	0.859
**Death in ICU**	1.299	0.650, 2.595	0.459
**Death in Hospital**	0.927	0.472, 1.824	0.827
**Duration of ICU stay**	1.006	0.993, 1.019	0.352
**Duration of hospitalization**	1.001	0.996, 1.006	0.634
**Mechanical ventilation**	0.758	0.499, 1.151	0.194
**Prior antimicrobial treatment**	1.150	0.806, 1.641	0.441
**Duration of hospitalization over 5 days before ICU admission**	1.351	0.958, 1.905	0.087
**Prior hospitalization**	1.225	0.800, 1.876	0.351
**Immunosuppression**	0.847	0.503, 1.425	0.531
**Infection**	0.895	0.609, 1.316	0.573

## Discussion

The present study is the first observational trial to evaluate use of the ATS criteria in predicting infection or colonization by MDROs in critically ill patients in China. The present single institution study found that MDROs were present in 38% of study patients at ICU admission. The NPV and PPV, determined by use of ATS guidelines for colonization, infection or pneumonia with MDROs at ICU admission, were very low in our study population. In addition, the predictive values differed among the different hospital departments. The proportion of MDROs in our population was much higher than previously reported for Europe and America [20]–[23]. Furthermore, MDROs were identified in 42% of patients from the emergency department, most of whom came from the community. This result highlights that antimicrobial resistance is a serious problem in China.

Previous studies demonstrated that antimicrobial resistance is increasing worldwide [8], [24], especially in developing countries. Bertrand and colleagues [25] reported that antimicrobial resistance among clinical isolates that were Gram-negative was more severe in the Asia-Pacific rim, Latin America, the Middle East, and Africa than in North America and Europe. Zhang et al. [26] reported that the mean percentage of antimicrobial resistance among community-acquired infections was as high as 26% (range: 15% to 39%) in China from 1999 to 2001, but was only 6% in the U.S.A. during the same time. We can suggest two reasons for the higher proportion of MDROs in our study. First, as a developing country, China has experienced a rapidly increasing rate of resistance to antimicrobial agents [26]–[29] due to the overuse of antibiotics [30]–[32]. Second, our population included more patients who were more seriously ill, and critically ill patients are more vulnerable to infections by MDROs [33].

In our patient population, the NPV and PPV (calculated from ATS guidelines) were both very low. These results differ from those of a previous study by Nseir et al., who reported that the PPV of the ATS criteria in predicting colonization or infection with MDROs at ICU admission was only 18% but that the NPV was 96% [17]. The lower overall NPV in our study (63.5%) may be related to the higher proportion of infection by MDROs in China. In fact, we found that patients from departments with 30% or less MDROs had a lower NPV than those from departments with 31–40% MDROs. In addition, the proportions of MDROs in patients from our surgery and medicine departments were 36.3% and 36.4%, and the NPVs were 65.3% and 71.9%. Taken together, this indicates that the rates of infection by MDROs can differ among different institutions and countries, and even among different departments within an institution.

Some previous studies have demonstrated that the ATS guidelines could help to facilitate appropriate antibiotic treatment at ICU admission. For example, Soo Hoo and colleagues found that implementation of ATS guidelines resulted in a higher rate of appropriate treatment and lower mortality [15]. Nachtigall et al. also demonstrated that adherence to standard operating procedure was associated with a shorter duration of treatment of first pneumonia episodes, shorter durations of mechanical ventilation, and shorter ICU stays [16]. These previous studies had satisfactory NPVs, but poor PPVs. Therefore, empirical use of antibiotics according to the ATS risk factors for MDRO appears to be effective. However, in our population, adherence to the ATS guidelines appeared to have less of an effect in improving the outcome of infected patients, as indicated by the low PPV and NPV. Use of these guidelines would lead to more than 60% of patients infected with non-MDRO being treated with broad-spectrum antibiotics. Thus, approximately one-third of the patients infected with MDROs would receive improper antibiotics if the physician selected antibiotics based on ATS guidelines.

Risk factors for colonization or infection with MDROs include prior antimicrobial treatment, prior hospitalization, and immunosuppression. In our study, there were very few patients who were residents in a nursing home or extended-care facility or who were given home infusion therapy or had home wound care. We did not separately analyze these patients because of the limited numbers. These risk factors are the same as the risk factors recommended by the ATS guidelines. However, we found that approximately one-third of patients who were colonized or infected with an MDRO had no risk factors according to the ATS guidelines. This is very different from the results of a previous study of a 30-bed ICU in France from December 2006 to December 2007 [17]. In our population, there was no statistical difference in the proportion of patients with MDRO risk factors in the MDRO and non-MDRO groups.

Previous research indicated that length of stay in the ICU was a risk factor for infection with an MDRO [34]. In the present study, the proportion of patients colonized or infected with an MDRO increased from 38% at ICU admission to 51% at ICU discharge. In addition, patients who stayed in the ICU for more than 7 days had an increased proportion of MDRO infection or colonization, from 42% at ICU admission to 76% at discharge (Fig. 2).

Our study has some limitations. First, this study was performed in a single center and was observational, so the results may not be relevant to ICU patients in general or even to other ICU patients in China. In particular, our findings were different from a previous study performed in France [17]. In the French study, the PPV was very low, but the NPV was very high. However, in our study, the PPV and NPV were both very low. This might be attributed to the higher proportion of drug resistant bacteria in our population, which may mean that patients carry MDROs even in the absence of risk factors, as assessed by the ATS guidelines. An interventional study is needed to determine the predictive value of the ATS guidelines. Previous studies in other hospitals demonstrated that the proportion of MDROs was high [26], suggesting that our results may applicable to certain other populations. Second, a large number of our patients with an infection or pneumonia were clinically diagnosed by a blood test, culture, imaging, and vital parameters. We did not perform semiquantitative or quantitative culturing. This may have affected the assessed value of the ATS guidelines in predicting colonization or infection. Further study is needed to resolve this limitation. Finally, we did not assess other factors related to colonization or infection by MDROs. In particular, we did not collect information regarding patient history before ICU admission. This means that we cannot explain why one-third of the patients who were colonized or infected with MDROs had no risk factors according the ATS criteria.

We conclude that there was a high rate of MDRO colonization and infection during the study period in our population. The value of the ATS criteria in predicting infection or colonization by MDROs was limited in critically ill patients at ICU admission in our hospital, but the predictive values differed among the different hospital departments. Additional studies with larger samples are required to develop criteria that can aid the early identification of patients infected with MDROs.

## Supporting Information

Table S1 (DOC)
