# Supplementary material for: Value of American Thoracic Society Guidelines in Predicting Infection or Colonization with Multidrug-Resistant Organisms in Critically Ill Patients
Source: PLoS One. 2014 Mar 19;9(3):e89687. doi: 10.1371/journal.pone.0089687 (PMC3960103; doi:10.1371/journal.pone.0089687)
Supplement: Table S1 — (DOC) [file pone.0089687.s001.doc]

**Supplementary Table 1.Number and percentage of MDROs in different departments.**

|  | **Overall** | **ICU admission** | | **ICU discharge** | | ***P*-value** |
| --- | --- | --- | --- | --- | --- | --- |
| All departments | 610 | 230 | 38% | 314 | 51% | <0.001* |
| Emergency | 176 | 71 | 40% | 101 | 57% | 0.001* |
| Neurosurgery | 30 | 10 | 33% | 21 | 70% | 0.004* |
| Thoracic surgery | 80 | 24 | 30% | 28 | 35% | 0.500 |
| General surgery | 101 | 35 | 35% | 43 | 43% | 0.248 |
| Urology surgery | 26 | 11 | 42% | 11 | 42% | 1.000 |
| Orthopaedics | 27 | 10 | 37% | 12 | 44% | 0.580 |
| Respiratory medicine | 20 | 10 | 50% | 13 | 65% | 0.337 |
| Neurology | 27 | 12 | 44% | 19 | 70% | 0.054 |
| Cardiology | 28 | 9 | 32% | 11 | 39% | 0.577 |
| Other | 95 | 38 | 40% | 55 | 58% | 0.014* |
